# Effects of food store quality on hibernation performance in common hamsters

**DOI:** 10.1371/journal.pone.0185913

**Published:** 2017-10-18

**Authors:** Carina Siutz, Matthias Nemeth, Karl-Heinz Wagner, Ruth Quint, Thomas Ruf, Eva Millesi

**Affiliations:** 1 Department of Behavioural Biology, Faculty of Life Sciences, University of Vienna, Vienna, Austria; 2 Department of Nutritional Sciences, Faculty of Life Sciences, University of Vienna, Vienna, Austria; 3 Department of Integrative Biology and Evolution, Research Institute of Wildlife Ecology, University of Veterinary Medicine, Vienna, Austria; Institute of Zoology, CHINA

## Abstract

Hibernating animals can adjust torpor expression according to available energy reserves. Besides the quantity, the quality of energy reserves could play an important role for overwintering strategies. Common hamsters are food-storing hibernators and show high individual variation in hibernation performance, which might be related to the quality of food hoards in the hibernacula. In this study, we tested the effects of food stores high in fat content, particularly polyunsaturated fatty acids (PUFAs), on hibernation patterns under laboratory conditions. Control animals received standard rodent pellets only, while in the other group pellets were supplemented with sunflower seeds. We recorded body temperature during winter using subcutaneously implanted data loggers, documented total food consumption during winter, and analysed PUFA proportions in white adipose tissue (WAT) before and after the winter period. About half of the individuals in both groups hibernated and torpor expression did not differ between these animals. Among the high-fat group, however, individuals with high sunflower seeds intake strongly reduced the time spent in deep torpor. PUFA proportions in WAT decreased during winter in both groups and this decline was positively related to the time an individual spent in deep torpor. Sunflower seeds intake dampened the PUFA decline resulting in higher PUFA levels in animals of the high-fat group after winter. In conclusion, our results showed that common hamsters adjusted torpor expression and food intake in relation to the total energy of food reserves, underlining the importance of food hoard quality on hibernation performance.

## Introduction

Animals can overcome periods of unfavourable environmental conditions by hibernation, characterized by strongly reduced metabolic rate and body temperature (T_b_) during multiday torpor bouts [1–5]. Most hibernating species exclusively rely on body fat reserves as metabolic fuel for hibernation, while others store food which can be used as external energy reserves [6–10]. The obvious advantage of saving energy during hibernation is accompanied by other benefits such as reduced water loss, parasite load, or predation risk [4, 5, 11–14]. Accordingly, it has been shown that individuals intensified torpor expression with increasing internal energy reserves [15, 16]. This overwintering strategy, however, might also implicate costs on an individual as torpor was found to be associated with oxidative stress [17], ischemia [18], shortening of telomeres [19], immune depression [20, 21], reduced synaptic efficacy [22], or impaired memory retention [23]. Hibernation could, therefore, be considered as a cost-benefit trade-off probably resulting in an adjustment of torpor expression in relation to available energy reserves [8]. Several studies on food-storing hibernators demonstrated that individuals strongly reduced torpor expression when food was abundant [7, 24–26]. Although torpor adjustment might be more pronounced in food-storing hibernators due to their greater energy-storing capacity [8], similar patterns were also found in fat-storing hibernators as individuals with higher body mass prior to hibernation reduced the depth of torpor expression or showed longer euthermic periods [27, 28].

Torpor expression, however, might not only be affect by the quantity, but also the quality of energy reserves. Polyunsaturated fatty acids (PUFA), which cannot be synthesized by mammals *de novo* and must be obtained from the diet, were found to play an important role in mammalian hibernation. Earlier studies focused on positive effects of PUFAs in general by maintaining membrane and depot fat fluidity via reducing lipid melting points [29, 30], but more recent studies suggested specific effects of certain fatty acids on hibernation [31, 32]. n-6 PUFAs, more precisely linoleic acid (LA, 18:2 n-6), were found to enhance hibernation performance [31–35], while torpor expression was reduced in response to n-3 PUFAs such as α-linolenic acid (ALA, 18:3 n-3) or docosahexaenoic acid (DHA, 22:6 n-3) [32, 35, 36]. On the other hand, PUFAs can also be considered as a valuable energy source and particularly food-storing hibernators could, therefore, adjust hibernation performance in relation to the PUFA content of their food hoards. Eastern chipmunks (*Tamias striatus*), for example, reduced torpor expression when provided with food supplements resembling the PUFA content of their natural diet, and further reduced torpor if the supplemented PUFA content exceeded that of the natural diet, although potential effects of food store size could not completely be excluded in this study [26].

Common hamsters (*Cricetus cricetus*) are an ideal model species to investigate effects of energy reserves and food composition on hibernation as they build up food stores and were found to show a high variation in hibernation performance [37–40]. In free-ranging hamsters, food caching activities in adult females were much more pronounced than in males, indicating larger food stores. Adult males on the other hand, had higher body fat proportions before winter compared with females [41]. Accordingly, females delayed hibernation onset while males expressed T_b_ patterns resembling those of obligate hibernators [37]. Despite regular, deep torpor bouts, common hamsters also express shallow torpor bouts lasting less than 24 hours, and exhibit a high flexibility in the use of these torpor types, particularly under laboratory conditions. Most importantly, they were found to adjust torpor expression in response to food stores. Hamsters facing unpredictable food store availability were more likely to hibernate and expressed shallow torpor more intensely compared to individuals with access to food stores [42].

In the present study, we manipulated food store quality, while size was standardized, in common hamsters under laboratory conditions by increasing the fat content and thus, the total energy of hoards. We provided two groups of hamsters with the same amount of food, but one group exclusively received standard laboratory food (pellets) while in the other group 25% of pellets were replaced by sunflower seeds, resulting in increased energy and also PUFA, particularly LA, content. We compared hibernation patterns between the two groups and also recorded the total pellets and sunflower seeds consumption during winter. Since in hibernators the composition of dietary fatty acids is reflected in the composition of depot fat and mitochondrial membranes [43], we additionally analysed PUFA proportions in white adipose tissue before and after the experimental period. If hamsters adjust hibernation performance according to food store quality, we would expect individuals of the high-fat group to reduce torpor expression, and by that potential costs of torpor, as they are energetically more flexible.

## Methods

### Ethical statement

The study was approved by the ethics committee of the Faculty of Life Sciences, University of Vienna (2014–008). All procedures performed on animals were permitted by the Austrian Ministry of Science, Research and Economy and the Ethical Committee for Animal Welfare (GZ BMWF-66.006/0039-II/3b/2013).

### Animals and housing conditions

We used 22 female common hamsters (aged 21 months; including 7 sibling pairs), obtained from a laboratory breeding colony in Strasbourg, France (Chronobiotron UMS 3415, Centre de Neurochimie). Animals were individually housed in transparent plastic cages (99 x 51.5 x 36 cm; Ferplast, Maxi Duna Multy) equipped with an artificial burrow system consisting of 3 boxes (each 23 x 16 x 14 cm; previous studies showed that hamsters used one box as nest box, one to store food, and another one was used for defecation) that were connected via plastic tubes. The lids of the boxes were removable to allow inspection of animals and food stores. The hamsters received food pellets (rodent standard pellets, Ssniff V2233, Ssniff Spezialdiäten GmbH, Soest, Germany; 3% fat, 17% protein, 13% fibre, 1.32 MJ/100g metabolizable energy content) and water *ad libitum* and were kept at 19±1°C under natural photoperiod length. To acclimatize the animals to the experimental conditions of 6±0.5°C ambient temperature and short photoperiod (6L:18D, lights on at 0800 h), we gradually reduced ambient temperature and photoperiod length starting 3 weeks prior to the onset of the experiment (19^th^ December 2014). This initial phase resembled natural conditions as burrow temperatures usually do not drop below 10°C before December in free-ranging hamsters. None of the animals hibernated before the onset of the experiment. Starting in late April, we continuously increased ambient temperature and photoperiod length until the end of the experiment (5^th^ May 2015), which resembled the date when the last hamsters at our field site had resumed above-ground activity. The duration of the experimental period was within the range of hibernation durations of free-ranging common hamsters as particularly adult females start to hibernate between late December and early January [37].

### Experimental design

The hamsters were assigned to 2 groups of 11 individuals (sibling pairs were not in the same group). We cleaned all cages shortly before the experiment started to ensure that previously stored food was removed and food store size provided for the experimental period was equal in all individuals during the experimental period. At the onset of the experiment, one group (control) received 2000g pellets (Ssniff V2233, 1.32 MJ/100g, 3.3g total fat/100g, 1.97g PUFA/100g consisting of 1.64g LA and 0.33g ALA) per individual to hoard, an amount known to be sufficient to survive the experimental period without the use of torpor [42]. The second group (high-fat, HF) received 1500g pellets (Ssniff V2233) mixed with 500g sunflower seeds (Dehner Natura, Dehner GmbH, Germany; 2.45 MJ/100g, 51.5g total fat/100g, 23.14g PUFA/100g consisting of 23.05g LA and <0.1g of all other PUFAs) [44]. This resulted in a total energetic value of 26.4 MJ provided for the control, and 32.03 MJ for the HF group. The food was placed at the entrance of the burrow system so that the hamster could cache and carry it inside the boxes. Body mass prior to the experimental period did not differ between the groups (control: 318±10 g, HF: 316±10 g; p = 0.88). Although sunflower seeds were about the same size and mass as pellets, they could have varied in their energetic content. Since the food in our study had to be palatable for at least 4 months, we used sunflower seeds instead of oil to avoid the risk of oxidation, which particularly applies to PUFA-rich oils. In addition, providing seeds to simulate the availability of high-quality food probably more closely reflected the natural situation as such food items, i.e. seeds, are also available and stored by free-ranging animals and might vary in their energetic content. At the end of the experiment, we thoroughly examined the cages and collected every remaining pellet and sunflower seed, which were then weighed to calculate the food intake during the experimental period.

### Hibernation patterns

Body temperature was recorded at 90-min intervals using temperature data loggers (iButtons, DS1922L-F5#, range: -40°C to +85°C, accuracy: ±0.5°C, Maxim Integrated Products International, Dublin, Ireland). The iButtons (coated in Elvax ethylene vinyl acetate resins, DuPont, and paraffin; gas-sterilised; potted mass: ~4.5 g) were implanted subcutaneously in the neck region (dorsal, between the scapulae) under isoflurane anaesthesia in a veterinary clinic about 1 month (12^th^ November 2014) prior to the experimental onset. This method has proved successful in this species [37]. The iButtons were removed in spring using the same technique.

**Fig 1:**
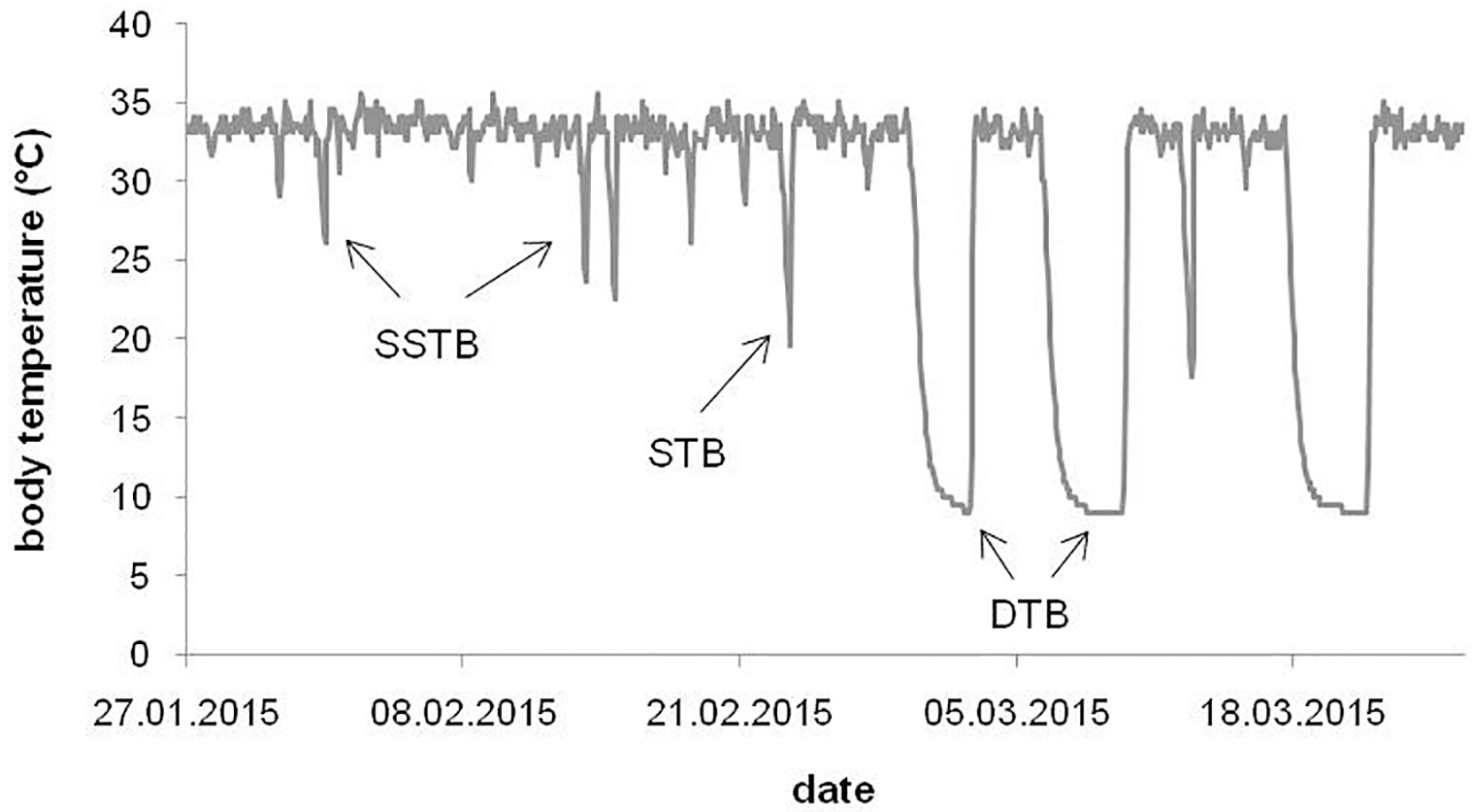
Representative section of a common hamster's T_b_ pattern under constant conditions demonstrating the three torpor types: Deep torpor bouts (DTB), short torpor bouts (STB), and short and shallow torpor bouts (SSTB).

Torpor was defined as the period when T_b_ was below 30°C. In addition to deep torpor bouts, characterized by T_b_ below 20°C (mean±SE: 10.1±0.2°C, n = 13) for longer than 24 h (2.7±0.1 d), we could identify 2 other types of torpor which are frequently expressed by common hamsters (Fig 1) [39]. First, short torpor bouts (STBs) with T_b_ drops below 20°C (18.5±0.2°C, n = 9) but a duration shorter than 24 h (12.9±0.4 h) and, second, short and shallow torpor bouts (SSTBs) in which T_b_ remained above 20°C (27.3±0.4°C, n = 21) for a few hours (4.7±0.3 h). In our study, STBs occurred relatively rarely in both groups (control: 1.8±0.3 bouts, n = 4; HF: 2.6±0.7 bouts, n = 5) and since STB and SSTB expression did not differ between the groups (p>0.11 for all parameters tested), we combined these 2 torpor types and hereafter refer to shallow torpor bouts (i.e. torpor bouts lasting less than 24h).

For both deep and shallow torpor bouts we analysed the number of bouts, the time spent in torpor (total duration of all torpor bouts; calculated in hours, expressed as days), bout duration (beginning from the sampling interval when T_b_ decreased below 30°C until it had reached 30°C again; calculated in hours, expressed as days), minimum T_b_ (lowest value of T_b_ during a torpor bout), and mean T_b_ (beginning from the sampling interval when T_b_ decreased below 30°C until it had reached 30°C again). In individuals that hibernated (i.e. showed deep torpor bouts), we additionally analysed the duration of the pre-hibernation period (days from the experiment onset to the onset of the first deep torpor bout), the duration of the post-hibernation period (days from the termination of the last deep torpor bout to the end of the experiment), and the hibernation duration (days from the onset of the first to the termination of the last deep torpor bout).

### Fatty acids

Fatty acids were analysed in total lipids from white adipose tissue (WAT). Subcutaneous WAT samples (~ 0.1–0.4 g) were taken from the interscapular region immediately before the insertion and removal, respectively, of the iButton and stored at -80°C until analyses. One individual of the HF group could not be sampled prior to the experiment due to virtually inexistent WAT at the sampling position. Lipid extraction was performed after Folch et al. [45] and sample preparation followed the protocol of Wagner et al. [46]. WAT samples (100 mg) were homogenised through a strainer (Cell STrainer—Falcon 100 μm Nylon) with a chloroform-methanol mixture (2:1, v/v; 2 x 3 ml) and 2 ml CaCl (0.05 M), washed with 2 ml distilled water, and the extracts were dried over N_2_ at 40°C. For fatty acid transesterification, 1 ml methanolic NaOH, containing butylated hydroxytoluene (BHT) to prevent oxidation, was added to the vaporised extracts and boiled at 100°C for 5 min. To obtain fatty acid methyl esters (FAMES), 1 ml 14% boran-triflourid-methanol (BF_3_) was added and again boiled at 100°C for 5 min. FAMES were extracted into 500 μl hexane four times, vaporised, and redissolved in hexane for gas chromatography analysis. FAMES were separated by a Rtx-2330 30 m x 0.25 mm i.d. silica column using an Auto-System-Gaschromatograph (Perkin Elmer, USA) equipped with a flame ionization detector (FID). FAMES (1 μl) were injected at a temperature of 250°C and detected at 270°C using helium as a carrier gas. Fatty acids were identified by a 37 component FAME Mix Standard ((Supelco, Bellafonte, USA) and peak integration was performed using the software TotalChrom Workstation 6.3.0 (PE Nelson, Perkin Elmer, USA).

Single fatty acids were summed up to calculate total percentages of n-3, n-6, and n-9 fatty acids. As we focused on PUFAs due to our experimental design of providing sunflower seeds, we restricted the analyses to total PUFA proportions, particularly as n-6 fatty acids (predominantly linoleic acid, C18:2 n6) accounted for 97.3% (n = 43) of total PUFAs found in WAT samples according to the gas chromatography analyses. All other PUFAs accounted for <1% of sample composition.

### Statistics

Statistical analyses were performed in R [47] additionally using the packages ‘nlme’ [48] for linear mixed models (LMEs) and ‘phia’ [49] for post-hoc analyses of significant interaction effects.

For group comparisons of hibernation performance we calculated LMEs for the parameters torpor bout duration, minimum T_b_, and mean T_b_ and included the parameter experimental group (control/HF) as a fixed effect and individual identity as a random effect to correct for repeated measurements. The parameters number and total duration of torpor bouts, pre-hibernation and post-hibernation period, and hibernation duration were compared using linear models. We each included experimental group as predictor variable and corrected for pre-hibernation body mass. Since pre-hibernation body mass had no effect in these analyses we omitted this parameter in Table 1 of the results section to simplify the presentation of these results. To analyse potential effects of food intake we calculated linear models for each hibernation parameter in both groups including total intake (i.e. pellets intake in the control and pellets plus sunflower seeds intake in the HF group) and pre-hibernation body mass as predictor variables, and calculated additional models for the HF group with pellets intake, sunflower seeds intake, and pre-hibernation body mass as predictors.

**Table 1:** Comparison of hibernation performance between individuals of the control and HF group.

Torpor type	response variable	group		*p* value
		control	HF	
deep	n	7	6	
	number	4 ± 0.8	5.5 ± 1.4	0.341
	total duration (d)	10.8 ± 2.5	15.5 ± 4.3	0.336
	mean duration (d)	2.6 ± 0.2	2.7 ± 0.2	0.652
	minimum T_b_ (°C)	10.4 ± 0.4	9.8 ± 0.3	0.116
	mean T_b_ (°C)	14.2 ± 0.4	13.8 ± 0.5	0.17
shallow	n	10	11	
	number	23.9 ± 7.9	39 ± 8.9	0.235
	total duration (d)	4.5 ± 1.2	6.8 ± 1.3	0.244
	mean duration (d)	0.2 ± 0.02	0.2 ± 0.02	0.3
	minimum T_b_ (°C)	26.6 ± 0.6	27.3 ± 0.5	0.336
	mean T_b_ (°C)	27.8 ± 0.3	28.1 ± 0.3	0.412

Values represent means ± SE.

*p* values were obtained from ANOVA (Type III) tables and are corrected for pre-hibernation body mass.

n: number of individuals expressing the respective torpor bouts

We applied an LME to analyse the proportions of total PUFAs (response variable) including sampling time (before/after the experimental period), group (control/HF) as well as their interaction as fixed effects and individual identity as a random effect. We calculated linear models to analyse potential effects of hibernation performance on PUFA change during the experimental period and included the parameters total duration of deep torpor bouts, total duration of shallow torpor bouts, and experimental group as predictor variables. We calculated an additional model that also included minimum T_b_ during deep and shallow torpor as well as experimental group as predictor variables as we thereby excluded individuals without deep torpor bouts from the statistical analyses. This model was fitted according to AICc (Akaike’s information criterion corrected for small sample size) reduction. This revealed that the parameter minimum T_b_ during shallow torpor had no effect and was, therefore, excluded from the final model, which included the parameters total duration of deep torpor bouts, total duration of shallow torpor bouts, minimum T_b_ during deep torpor, and experimental group as predictor variables. Finally, we calculated a linear model for effects of sunflower seeds intake (predictor variable) on PUFA change (response variable) among individuals of the HF group. Model residuals were tested for normality using Shapiro–Wilk tests and for homoscedasticity using Levene-tests. *P* values were obtained from ANOVA (Type III) tables (package 'car', [50]). Within the HF group, the relationship between pellets and sunflower seeds intake was analysed using Pearson correlation. Significance level was set at *p* ≤ 0.05. Results are presented as means ± SE.

## Results

### Hibernation performance

About half of the individuals in each group hibernated (i.e. showed deep torpor bouts), and number, duration, and T_b_ of deep torpor bouts did not differ between the groups (Table 1). In addition, we found no differences in the duration of the pre-hibernation period (control: 84 ± 10.2 d, HF: 89.6 ± 10 d, p = 0.769) as well as post-hibernation period (control: 11.3 ± 1 d, HF: 14.9 ± 6 d, p = 0.672), resulting in similar hibernation durations (control: 41.7 ± 11.1 d, HF: 32.6 ± 6.2 d, p = 0.511). One individual of the control group remained continuously euthermic throughout the experimental period. All other individuals showed at least shallow torpor bouts (lasting <24 h), and the expression of these bouts was similar in both groups (Table 1). The number and total duration of deep torpor bouts was not related to the number or total duration of shallow torpor bouts (p>0.1 in both cases). We additionally combined the two types of torpor and calculated the total number and time spent in torpor, and again found no differences between the groups (number: control: 26.7 ± 7.7 bouts, n = 10, HF: 42 ± 8.3 bouts, n = 11; p = 0.205; total duration: control: 12.1 ± 2.4 d, HF: 15.2 ± 3 d, p = 0.42).

### Food intake, pre-hibernation body mass, and hibernation performance

**Fig 2:**
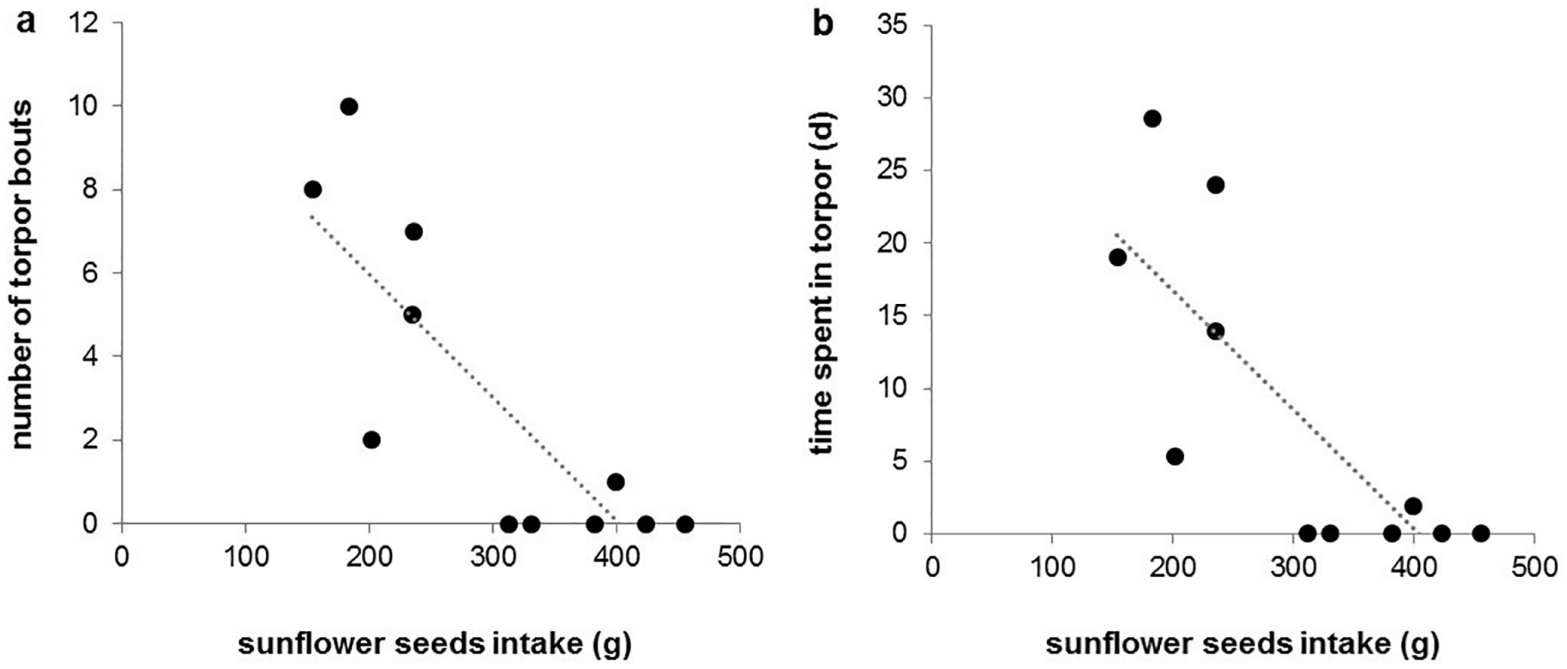
Effects of sunflower seeds intake on (a) number and (b) time spent in deep torpor in individuals of the HF group.

The total amount of food consumed during the experimental period had no effect on hibernation performance, neither in the control (Table 2) nor in the HF group (p≥0.1 in all cases). Pellet intake did not differ between the groups (control: 94.1 ± 1.6%, n = 11; HF: 95 ± 2%, n = 11; Student’s *t* test: p = 0.714). However, among individuals of the HF group, we found a relatively high variation in sunflower seeds intake, ranging from 154 g (30.8%) to 455 g (91%). Pellets and sunflower seeds intake were not related (r = -0.145, p = 0.67). When analysing effects of pellets and sunflower seeds intake, we found that pellet intake did not affect hibernation performance whereas high sunflower seeds intake reduced the number of deep torpor bouts and correspondingly, the total time spent in deep torpor (Table 2, Fig 2). Mean duration and T_b_ of deep torpor bouts were not affected. In general, sunflower seeds intake and the corresponding energy uptake were higher in individuals that did not enter deep torpor (381 ± 27 g and 9.3 ± 0.7 MJ, n = 5) compared to hibernating ones (235 ± 35 g and 5.7 ± 0.9 MJ, n = 6; Student’s *t* test: p = 0.010). Regarding shallow torpor bouts, we found that individuals with a high sunflower seeds intake showed shorter bouts and expressed them at higher T_b_ (Table 2). When combining both deep and shallow torpor bouts, we found that high sunflower seeds intake resulted in reduced time spent in torpor, although the overall number of torpor bouts was not affected (Table 2).

**Table 2:** Effects of food intake (pellets intake in control group; pellets and sunflower seeds intake in HF group) and pre-hibernation body mass on hibernation performance.

Torpor type	group	control		HF		
	response variable	body mass	pellets	body mass	pellets	sunflower
deep	number	1.383	-0.054	0.415	0.068	-3.678 **
	total duration (d)	1.271	-0.280	0.490	0.242	-3.286 *
	mean duration (d)	0.755	-1.835	-0.610	1.191	-1.543
	minimum T_b_ (°C)	-0.617	1.644	0.005	0.023	0.462
	mean T_b_ (°C)	-0.245	1.332	0.501	-0.637	1.675
shallow	number	-3.257 *	0.308	-0.339	1.345	1.279
	total duration (d)	-4.368 **	-0.627	0.001	1.399	0.757
	mean duration (d)	1.362	0.017	-0.953	1.352	-3.283 *
	minimum T_b_ (°C)	-1.200	-0.310	2.044	-1.342	4.488 **
	mean T_b_ (°C)	-1.264	-0.345	2.546 *	-1.582	5.021 **
both	number	-2.872 *	0.282	-0.316	1.412	0.998
	total duration (d)	0.218	-0.281	0.617	1.302	-3.597 **

Data are given as *t* values obtained from coefficient tables and significant effects are indicated by asterisks (*p≤0.05, **p≤0.01).

Torpor type ‘both’: deep and shallow torpor bouts combined

Among individuals of the control group, higher pre-hibernation body mass reduced the number and time spent in shallow torpor and also decreased the overall number of torpor bouts (i.e. deep and shallow bouts combined; Table 2). In the HF group, pre-hibernation body mass only affected mean T_b_ during shallow torpor bouts in that heavier individuals expressed these bouts at lower T_b_ (Table 2).

### PUFA status

**Fig 3:**
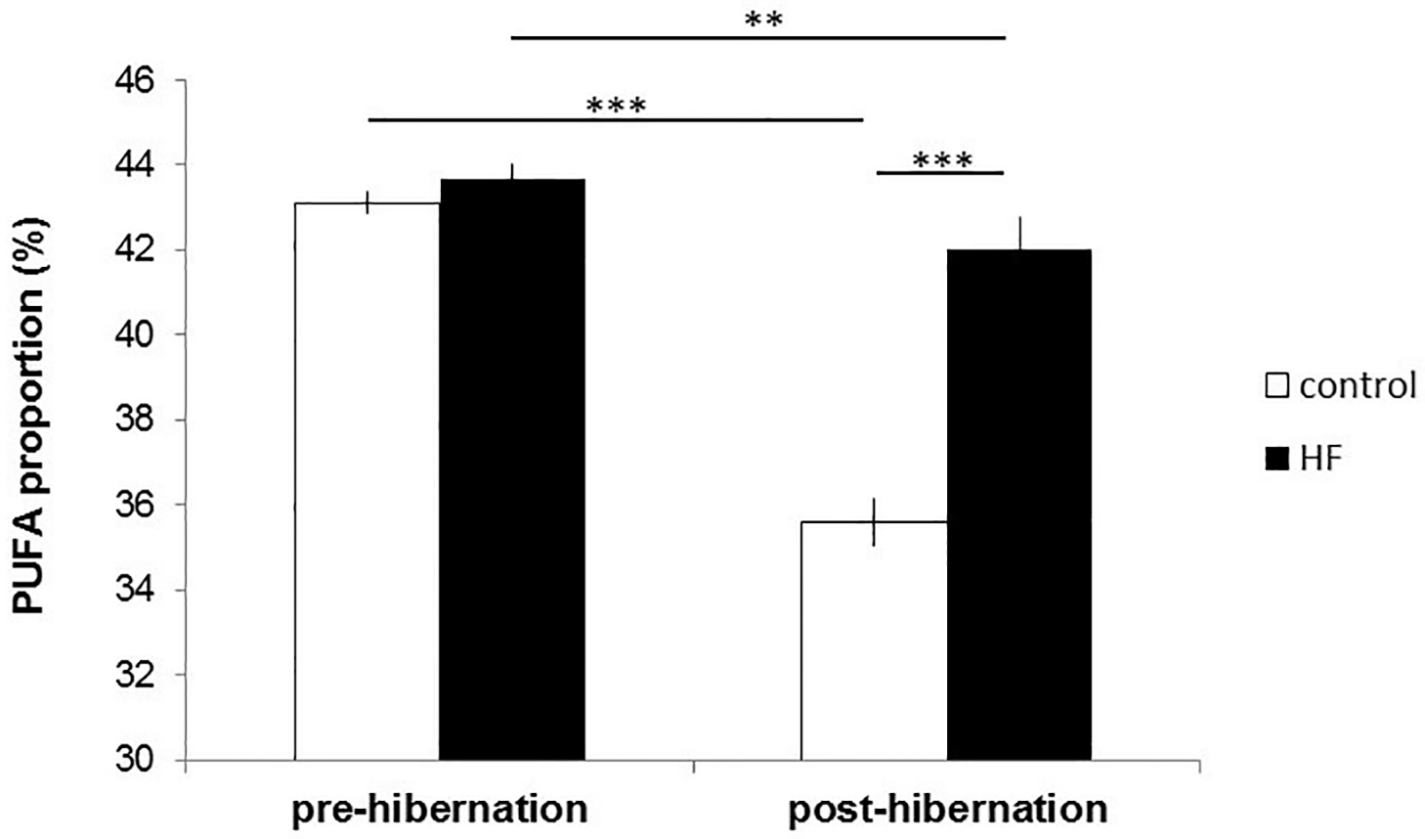
Proportions of total PUFA in WAT before and after the experimental period in animals of the control and HF group. ** p≤0.01, ***p≤0.001.

**Fig 4:**
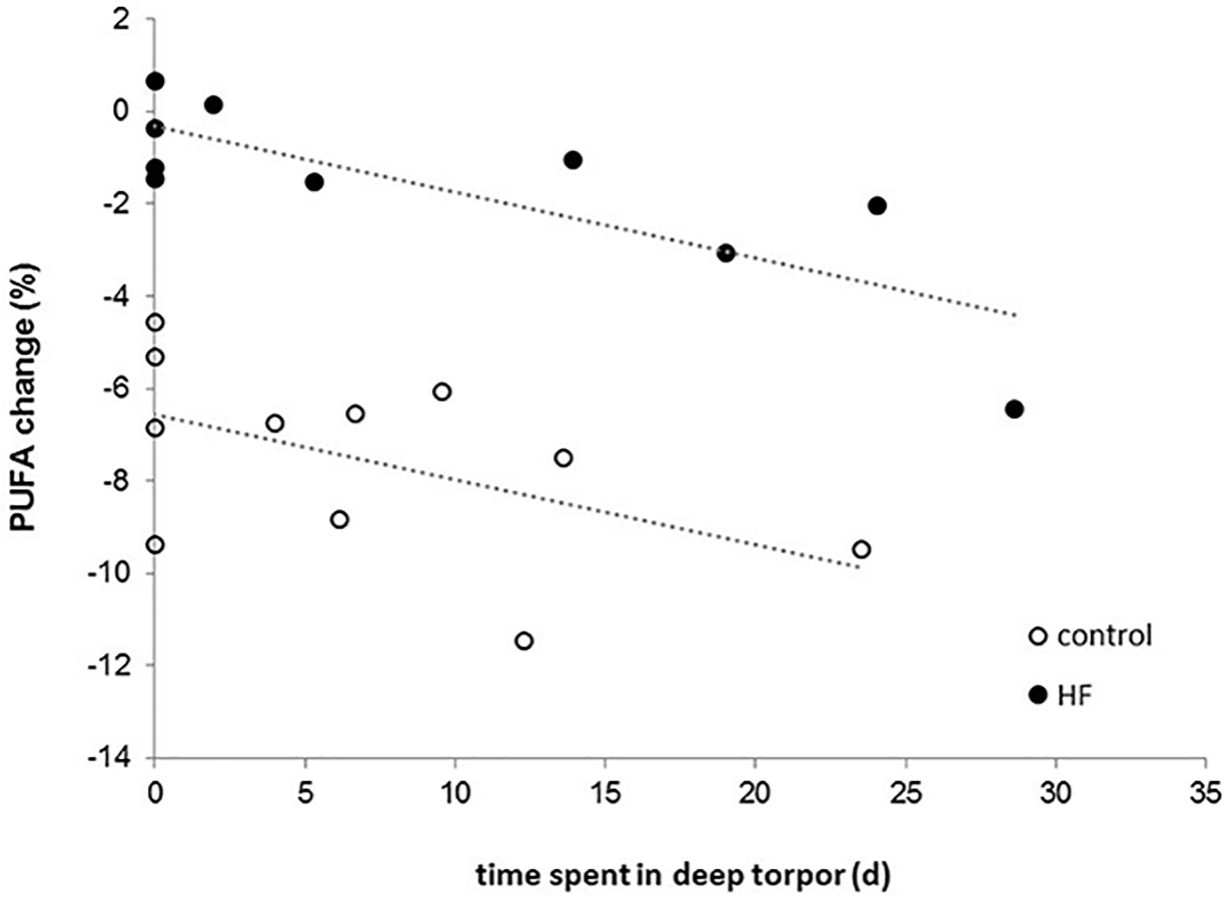
Effect of time spent in deep torpor on PUFA change in individuals of the control and HF group.

**Fig 5:**
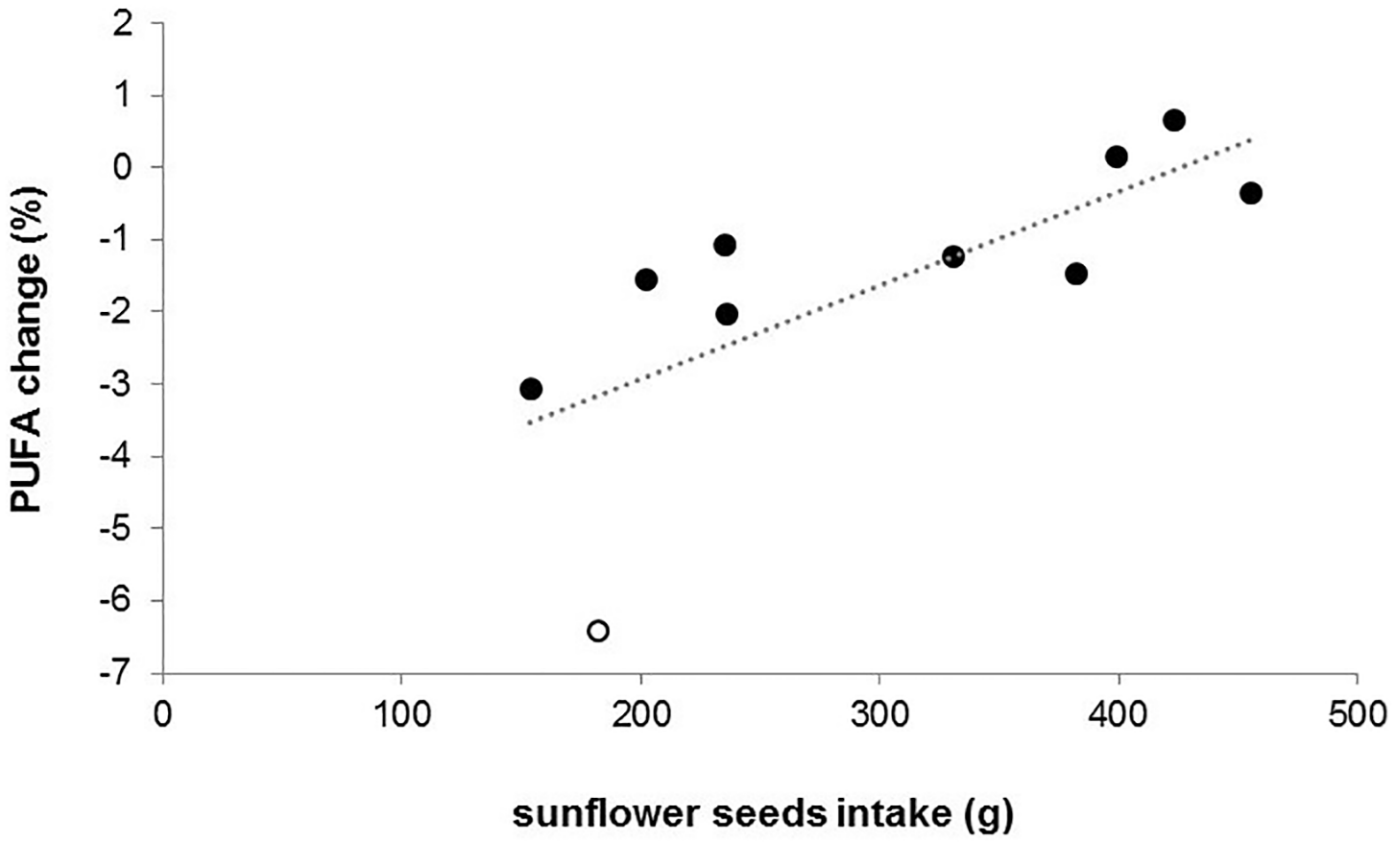
Effect of sunflower seeds intake on PUFA change in individuals of the HF group. Open circle: individual with most deep torpor bouts (n = 10) and longest time spent in deep torpor (28.6 d).

The proportions of total PUFAs in WAT were significantly affected by sampling time (before/after the experimental period), group, and their interaction (sampling time: F_1,19_ = 115.56, p<0.0001; group: F_1,20_ = 30.78, p<0.0001; sampling time x group: F_1,19_ = 45.49, p<0.0001; Fig 3). PUFAs were similar in both groups at the onset of the experiment (χ^2^ = 0.57, p = 0.452) and decreased during the experimental period in both groups (control: χ^2^ = 169.96, p<0.0001; HF: χ^2^ = 7.63, p = 0.006). This decline was more pronounced in individuals of the control group, resulting in lower PUFA proportions at the end of the experiment compared to individuals of the HF group (χ^2^ = 81.11, p<0.0001; Fig 3). Hibernation performance significantly affected the PUFA decline in both groups (Fig 4): the more time an individual spent in deep torpor, the stronger was the decrease in PUFAs (F_1,16_ = 10.25, p = 0.006) while the time spent in shallow torpor had no effect (F_1,16_ = 0.31, p = 0.585). This model also revealed a significant main effect of group (F_1,16_ = 10.25, p<0.0001) showing again that the PUFA decline was stronger in control than HF animals (Fig 4). When including minimum T_b_ during deep torpor in the model, and by that excluding non-hibernating individuals, we still found that the decrease in PUFAs was stronger the more time an individual spent in deep torpor (F_1,8_ = 17.09, p = 0.003), while the time spent in shallow torpor had no effect (F_1,8_ = 3.45, p = 0.1). Additionally, individuals expressing higher minimum T_b_ during deep torpor bouts had a stronger PUFA decrease (F_1,8_ = 6.86, p = 0.031). The PUFA decline in hibernators was again stronger in the control than in the HF group (F_1,8_ = 63.43, p<0.0001). Finally, increased sunflower seeds intake among individuals of the HF group resulted in lower PUFA decrease (F_1,8_ = 8.71, p = 0.018; Fig 5).

### Body mass change

Body mass after the experimental period did not differ significantly between the groups (control: 302 ± 11 g, HF: 321 ± 7 g; p = 0.176). Individuals of the control group lost on average 4.6 ± 3.6% of their initial mass, while individuals of the HF group gained on average 1.9 ± 2.2%, however this difference was not significant (Student’s *t* test: p = 0.141).

## Discussion

We manipulated food hoard quality in common hamsters under laboratory conditions by adding sunflower seeds to the standard pellets diet in one group and compared hibernation patterns to that of individuals receiving standard pellets exclusively. The amount of hoarded food, therefore, was equal in both groups, but the total energy of food stores differed. The availability of large food stores in general appeared to reduce torpor expression as only about half of the individuals in both groups hibernated (i.e. showed deep torpor bouts) and among those, the hibernation period was strongly shifted towards the end of the experimental period resulting in relatively short hibernation durations. Similar results were found in a recent study under laboratory conditions where hamsters with access to food stores were less likely to hibernate compared to individuals facing unpredictable food availability by being provided with daily food portions which prevented them from accumulating a food store [42]. Furthermore, in free-ranging common hamsters, adult females were found to delay hibernation onset compared to adult males and juveniles of both sexes, which is presumably related to larger food stores in adult females [37, 41]. Given the long duration of the pre-hibernation period and a rather short post-hibernation period in our study, hamsters most likely consumed their food primarily before the onset of hibernation and not thereafter. The shift of hibernation towards the end of winter indicates that hamsters relied as long as possible on food stores combined with less energy-saving shallow torpor, which occurred within a few days after the experimental onset, and switched to the highly energy-saving deep torpor by the time their food stores were close to depletion. This would be in line with the suggested trade-off between costs and benefits of torpor in that individuals adjust torpor expression in relation to the availability of energy reserves [8].

We found no differences between the groups in torpor expression, but the proportion of sunflower seeds intake and T_b_ patterns within the HF group varied highly among individuals. High sunflower seeds intake resulted in avoidance of deep torpor and, hence, reduced time spent in deep torpor, as well as shorter shallow torpor bouts expressed at higher T_b_. Such negative effects on hibernation were not found for pellets or total food intake, respectively, in this group. Thus, not food intake *per se* affected torpor expression but only that of sunflower seeds, i.e. food with high energy density. Despite the high variation in sunflower seeds intake, the results met our expectations by reflecting the cost-benefit trade-off of torpor expression. Those individuals that consumed large amounts of sunflower seeds presumably perceived them as energetically highly valuable, almost abandoned deep torpor, and additionally decreased shallow torpor expression, by that reducing potential costs of torpor. Non-hibernating hamsters within this group consumed more sunflower seeds than hibernating ones and, therefore, compensated their increased energetic expenditure by high caloric intake. Although individual food preferences cannot be completely excluded, it seems unlikely that this accounted for the variation in sunflower seeds intake because the hamsters were familiarized with sunflower seeds for a short period several months prior to the experiment and all animals well accepted these food items. The reason for this variation, therefore, remains unclear. Similar to the HF group, pellets intake had no effect on hibernation performance in control animals. However, control individuals with higher pre-hibernation body mass spent less time in shallow torpor than lighter ones, which to some degree also fits to the trade-off mentioned above. Among animals of the HF group, effects of pre-hibernation body mass only played a minor role indicating that internal energy reserves might be less important if high-quality food stores are available. Regarding potential effects on body condition after the experimental period we found that individuals of the HF group on average gained body mass while control animals lost body mass over winter, although these changes were only marginal and not significant. A balanced interplay between pre-hibernation body mass, food intake, and hibernation performance might account for these findings and would reflect various successful overwintering strategies in common hamsters.

Several studies demonstrated that increased amounts of PUFAs, more precisely linoleic acid (LA), were beneficial for hibernation as elevated LA concentrations in diets or WAT promoted hibernation since individuals were more likely to enter torpor, prolonged torpor bout duration, or tolerated lower T_b_ during torpor [e.g., 33, 34, 35]. These effects, however, were more pronounced at low ambient temperatures [e.g., 34]. One prominent effect of LA is the increase in the activity of the Ca^2+^-Mg^2+^ pump in the sarcoplasmic reticulum of the heart (SERCA 2a), enabling a proper Ca^2+^ handling of myocytes and by that protecting the heart from arrhythmia at low T_b_ [32, 35]. Thus, with increasing SERCA activity hibernating animals can tolerate lower T_b_. Sunflower seeds intake in our study, therefore, could have been beneficial in terms of SERCA activity as LA was the predominant PUFA in WAT samples. Furthermore, positive effects of LA on hibernation performance might be attributed to a limited availability of LA in the natural diet of hibernators as PUFA composition varies among plant species [51–53]. This would be an explanation for the absent group differences in torpor expression in our study, because pre- and post-hibernation PUFA levels were mostly above the range of those found in other herbivorous hibernators [30], indicating that LA was not a limited resource in our study. This would also exclude that a lack of PUFAs prior to hibernation, which was also found to reduce torpor expression [54, 55], was the reason for either the long pre-hibernation period or deep torpor avoidance, respectively. Furthermore, LA seemed not to be limited in the natural diet of common hamsters as individuals inhabiting agricultural and urban areas foraged on LA-rich food such as oats, rapeseed, wheat, acorn or hazelnut (Roswag et al., under review). If LA availability is not limited in the diet of a species, positive effects on hibernation are probably not to be expected, or hibernation can even be abandoned because of the high energetic value. Our results, therefore, indicate that hamsters with high sunflower seeds intake did not hibernate because they simply did not need to, due to the availability of energy-rich food stores [26].

Interestingly, proportions of PUFA in WAT decreased during winter in both groups. This is contrary to other studies where PUFAs, particularly LA, were found to increase over winter as hibernators usually selectively retain PUFAs and preferentially oxidize monounsaturated or saturated fatty acids during hibernation [51, 55–60]. The pattern found in our study was more similar to that of non-hibernating mammals and humans with short and unsaturated fatty acids being more readily metabolized [61, 62]. One explanation could be that LA availability in the diet indeed was not limited and hamsters could afford to oxidize PUFAs, or LA specifically, during hibernation. Although individuals in our study could have used PUFAs during the long prehibernation period as metabolic fuel to support thermoregulation at cold ambient temperatures [61], the strongly negative effect of deep torpor expression on PUFA levels rather supports the assumption that PUFAs are mobilized during hibernation in common hamsters. The PUFA decline was stronger the more time an individual spent in deep torpor, but was not affected by shallow torpor expression. Lower T_b_ during deep torpor, however, dampened the PUFA decline which might be simply due to stronger reduced metabolic rates at lower T_b_. Additionally, sunflower seeds intake attenuated the drop in PUFAs resulting in a less pronounced decrease during winter and thus, higher PUFA levels after the experimental period in HF compared to control animals. The positive effect of sunflower seeds intake and the negative effect of deep torpor on PUFA change over winter is further supported by our findings that PUFAs decreased in all individuals except two of the HF group which showed more or less unchanged PUFA levels (+0.15% and +0.66%, respectively) and were among those with the highest sunflower seeds intake (80% and 85%, respectively) and, hence, marginal deep torpor expression (one and no bout, respectively). Correspondingly, the individual with most deep torpor bouts and the longest time spent in deep torpor had only a moderate sunflower seeds intake and showed the strongest PUFA decline. Further studies, both in the lab and the field, are required to not only confirm this pattern but also to shed light on the mechanisms of fatty acid mobilization in this hibernator.

In conclusion, our results highlight a remarkable flexibility in hibernation performance and reflect different overwintering strategies in common hamsters. Individuals had the same pre-hibernation conditions but differently responded to energy reserves available for hibernation and adjusted torpor expression and food intake in relation to food store quality and pre-hibernation body mass. PUFAs appeared to be metabolized in this hibernator, but an increased dietary PUFA intake reduced this decline during hibernation resulting in higher PUFA levels after winter in individuals of the HF group compared to control animals. This could be beneficial as PUFAs are precursors for prostaglandins and thus, high availability of PUFAs in depot fats could ultimately improve reproductive success [63]. The role of PUFAs in reproductive performance, however, remains to be investigated in common hamsters.

## Supporting information

S1 File Dataset. (XLSX)
